# Comment on ‘Toxicities associated with immune checkpoint inhibitors: A systematic study’

**DOI:** 10.1097/JS9.0000000000000478

**Published:** 2023-05-18

**Authors:** Mohammad-Salar Hosseini, Farid Jahanshahlou

**Affiliations:** a Hematology and Oncology Research Center; b Research Center for Evidence-Based Medicine, Iranian EBM Center: A Joanna Briggs Institute Center of Excellence; c Aging Research Institute, Tabriz University of Medical Sciences, Tabriz, Iran

*Dear Editor,*

Kong *et al.* ^1^ have performed an interesting systematic review of immune checkpoint inhibitor-related clinical toxicities. We aim to address some issues regarding this recently published study.

First, we must highlight that the immune checkpoint inhibitors are not limited to the PD-1/PD-L1 (programmed cell death-1 and programmed cell death-ligand 1) pathway, and acknowledging the article’s title, a systematic study of all immune checkpoint inhibitor-associated toxicities is expected. However, although CTLA-4 has been mentioned among the study’s control group, no keyword addressing this pathway has been included in the search strategy, and the study design has only focused on the PD-1/PD-L1 pathway. The lack of keywords addressing the CTLA-4 pathway, despite the presence of drugs such as ipilimumab and tremelimumab that regulate this pathway, questions the accuracy of the study design. Moreover, the comparison between PD-1/PD-L1 and CTLA-4 inhibitors in the ‘Results’ section is methodologically incorrect, as the pool of CTLA-4 patients has definitely been formed from an insufficient number of studies due to the lack of proper representative keywords and the obviously missed studies in the final pool. Therefore, the selected studies are not representative of the title or the primary aim of the study.

Second, the search strategy seems improper. Apart from using incorrect terms like ‘programmed cell death 1 ligand 1’ among the main keywords (which has also been repeated in the main text of the article), the search strategy is accompanied by some structural flaws. Based on the Preferred Reporting Items for Systematic Reviews and Meta-Analyses (PRISMA) 2009 statement and guideline, the authors should ‘present full electronic search strategy for at least one database, including any limits used, such that it could be repeated’, which has recently been updated in PRISMA 2020 to ‘present the full search strategies for all databases, registers and websites, including any filters and limits used’^2,3^. The authors have not presented the full search strategies for any of the databases; the keywords representing the PD-1/PD-L1 pathway have been mentioned in the main text and the supplementary file, but the combination of the keywords that were used in conducting the systematic search is unknown, which leads to irreproducibility of the results and violates the PRISMA statement. We tried to repeat the systematic search in PubMed and Embase (no search strategy was provided for Web of Knowledge and China National Knowledge Infrastructure), and since the combination of the keywords representing the PD-1/PD-L1 pathway was unknown, we used the Boolean operator ‘OR’ to maximize the sensitivity and obtain the broadest strategy. The other keywords were used the same as the authors. Our search resulted in less than 20 results, none of which were relevant to the study topic or included in the final pool. Since the authors’ search has resulted in a sum of 444 results in all databases, we believe that the search strategies presented by the authors might be incomplete or incorrect. More importantly, the possible flaws in the search strategy have led to the missing of several vital studies^4,5^, which affect the ultimate findings of this review.

Third, there are some inconsistencies between the study and the registered protocol (CRD42019135113), such as the omission of the Scopus database from the search strategy and abandoning the effort to exclude the abstracts when in fact, the authors had included the abstracts by manual search. PRISMA guidelines always stress the importance of registering the study protocol in advance. The key reason for this is to help readers and evidence consumers assess the study against reporting bias and selective representation of findings.

The field of cancer immunotherapy is rapidly evolving; new targets are introduced and new drugs are approved every day. We sincerely appreciate the authors’ willingness to update the study, as mentioned in the ‘Strengths and Limitations’ section. Since it has been over 3 years since the study was conducted and many more drugs have been approved and studied in this field, the results might be uncertain in their current state, and the authors are recommended to update the study as soon as feasible.

We want to express our appreciation to the authors for their valuable and insightful contribution, which we believe would greatly benefit the scientific community. We recommend that the authors take into consideration the mentioned issues to ensure the quality and reliability of this research endeavor.

## Ethical approval

Not applicable.

## Consent

Not applicable.

## Sources of funding

None.

## Author contribution

M.S.H.: conceptualization, data curation, and writing – original draft; F.J.: data curation and writing – review and editing.

## Conflicts of interest disclosure

There are no conflicts of interest.

## Research registration unique identifying number (UIN)

Not applicable.

## Guarantor

Mohammad-Salar Hosseini.

## Provenance and peer review

Not commissioned, externally peer-reviewed.

## Data availability statement

Not applicable.
